# An origin identification model for labeling of shiitake (*Lentinula edodes*)

**DOI:** 10.1038/s41538-021-00085-4

**Published:** 2021-02-02

**Authors:** Ill-Min Chung, Yun-Ju Kim, Chang Kwon, Hee-Sung Moon, Jae-Gu Han, Won-Sik Kong, Seung-Hyun Kim

**Affiliations:** 1 Department of Crop Science, College of Sanghuh Life Science, Konkuk University, Seoul, 05029 Republic of Korea; 2 National Institute of Horticultural and Herbal Science, Rural Development Administration, Eumseong, 27709 Republic of Korea

**Keywords:** Fungal biology, Mass spectrometry

## Abstract

With the increasing globalization of the food trade across countries and continents, reliable identification of the geographical origin of products is critical. In this study, we describe the limitations of the current origin labeling system for non-soil-based agricultural products and suggest alternative strategies for the identification of the geographical origin of such products. An origin identification model based on stable isotope ratio analysis combined with discriminant analysis is used to evaluate the similarities and dissimilarities between domestic and foreign shiitake mushrooms, including Chinese inoculated sawdust blocks and Chinese origin. The results show a classification sensitivity of 92.0%, classification specificity of 91.5%, and overall accuracy of 93.5%. In particular, δ^15^N was the most important isotope marker for the identification of the origin of shiitake mushrooms. Hence, the current origin labeling system for mushroom species has to be revised to establish fair trade and avoid improper origin labeling in the global shiitake market.

## Introduction

With the recent expansion of globalized food markets, reliable geographical identification is essential to avoid mislabeling of foodstuffs^1,2^. The products subjected to the rules of geographical identification for trade between European Union (EU) countries include cheeses, beers, and meats^3^. The Japanese Agricultural Standard Law also mandates labeling of the geographic origin of foodstuffs to protect consumers and farmers from fraudulent labeling or imitation^4,5^.

Within the past two decades, global mushroom production has increased substantially^6,7^. Owing to their unique flavor and taste, shiitake mushrooms (*Lentinula edodes*) are the second most popularly consumed mushroom worldwide and account for ~22% of the total global mushroom production^8–10^. A sawdust block method was recently popularized for the supply of high-quality shiitake to consumers owing to its advantages over traditional methods^11^. In this method, a block composed of oak sawdust, straw, corn cobs, and additional supplements is used for cultivation^12–15^. In the United States of America (USA), gypsum, manure, cottonseed hulls, corn cobs, and wheat straw are commonly used in the sawdust blocks^14^. Oak tree sawdust and rice bran are typically used for the production of shiitake mushrooms via the sawdust block method in Korea; however, the method has not yet been standardized.

In Korea, shiitake mushrooms available in the market originate from domestic sources (41%), imported Chinese inoculated sawdust blocks (23%), and China (36%); the quantity of imported Chinese inoculated sawdust blocks for shiitake mushroom production has recently increased^16^. Subsequently, labeling issues regarding the geographical origin of shiitake mushrooms have recently emerged in Korea. According to the Origin Labeling Act (to be implemented in December 2020), origin labeling of shiitake mushrooms depends on the duration of sawdust block preparation, inoculation, and cultivation in a specific country. Moreover, the rules adopted by the World Trade Organization suggest that labels should indicate the country in which the commodity was fully obtained or, if more than one country was involved in the production of the commodity, the country in which the final substantive transformation was made. Consequently, if the cultivation period of shiitake mushrooms produced using imported Chinese inoculated sawdust blocks in Korea is longer than the sawdust block preparation/inoculation period in China, they should be labeled as domestic (Korean origin), not imported (Chinese origin). Similarly, origin labeling issues for shiitake mushrooms were also reported in the USA^17^, resulting in competition issues with many American farms. Accordingly, methods for the reliable determination of shiitake mushroom origin are needed.

Despite a lack of standardized methods, various analytical approaches (i.e., separation, spectroscopy, spectrometry, DNA, and sensory analysis) combined with chemometrics have been extensively studied for application in the identification of the accurate geographical origin of foodstuffs (including cereals, beverages, vegetables, meats, and wines) for the past two decades^18–21^. However, few reports have described the identification of the origin of mushroom species. For example, in Korea, the identification of domestic shiitake mushrooms compared with foreign shiitake mushrooms relies mostly on the morphological features (i.e., cap shape, size, and homogeneity) of the fruiting body or the documents associated with import/export. Thus, a reliable analytical method for the Agricultural Food Country of Origin Labeling (COOL) system is urgently needed for all stages associated with the production, distribution, consumption, and processing of shiitake mushrooms^22^.

In general, because living organisms in nature display unique isotopic features by physical, chemical, or microbial isotopic fractionation according to independent environmental and geological factors, stable isotope ratio analysis (SIRA) combined with chemometrics is emerging as a reliable and promising tool for the COOL system of agricultural products^23–29^. In cases of photosynthetic- or soil-based agricultural products, δ^13^C is mainly associated with the photosynthetic system (C3 versus C4)^30^, whereas δ^15^N is affected by regional agricultural practices, N availability, and isotopic fractionation by chemical, physical, microbial, and nutritional conditions in local soil^31,32^. Additionally, δ^18^O is influenced by geoclimatic conditions (i.e., altitude, latitude, continental effects, relative humidity, temperature, and amount of precipitation)^33^. δ^34^S is mostly related to soil geological properties and is somewhat affected by anthropogenic activities and sea-spray effects^23,28,34,35^. However, unlike common soil-based agricultural products, most edible mushroom species, including shiitake mushrooms, are produced using the cultivation substrate system in green houses. Additionally, mushrooms are nonphotosynthetic living organisms, and the sawdust block and the environment, such as temperature, humidity, and water/nutrient availability during the mushroom growth period, may affect the δ^13^C, δ^15^N, δ^18^O, and δ^34^S features via certain bio-physiological isotopic fractionations^36,37^. None of systematic studies have been reported the impact of the cultured medium versus cultivation environments on bulk isotope ratios in mushroom so far.

To our knowledge, however, with the exception of sensory determination based on the morphological features of the shiitake fruiting body, few reliable studies have reported discrimination of the origin of shiitake mushrooms consumed in Korea. Here, we examined the feasibility of geographical identification of shiitake mushrooms consumed in Korea using SIRA and established an approach for the geographical traceability of shiitake mushrooms.

## Results

### Cultivation methods for shiitake mushrooms

**Fig. 1:**
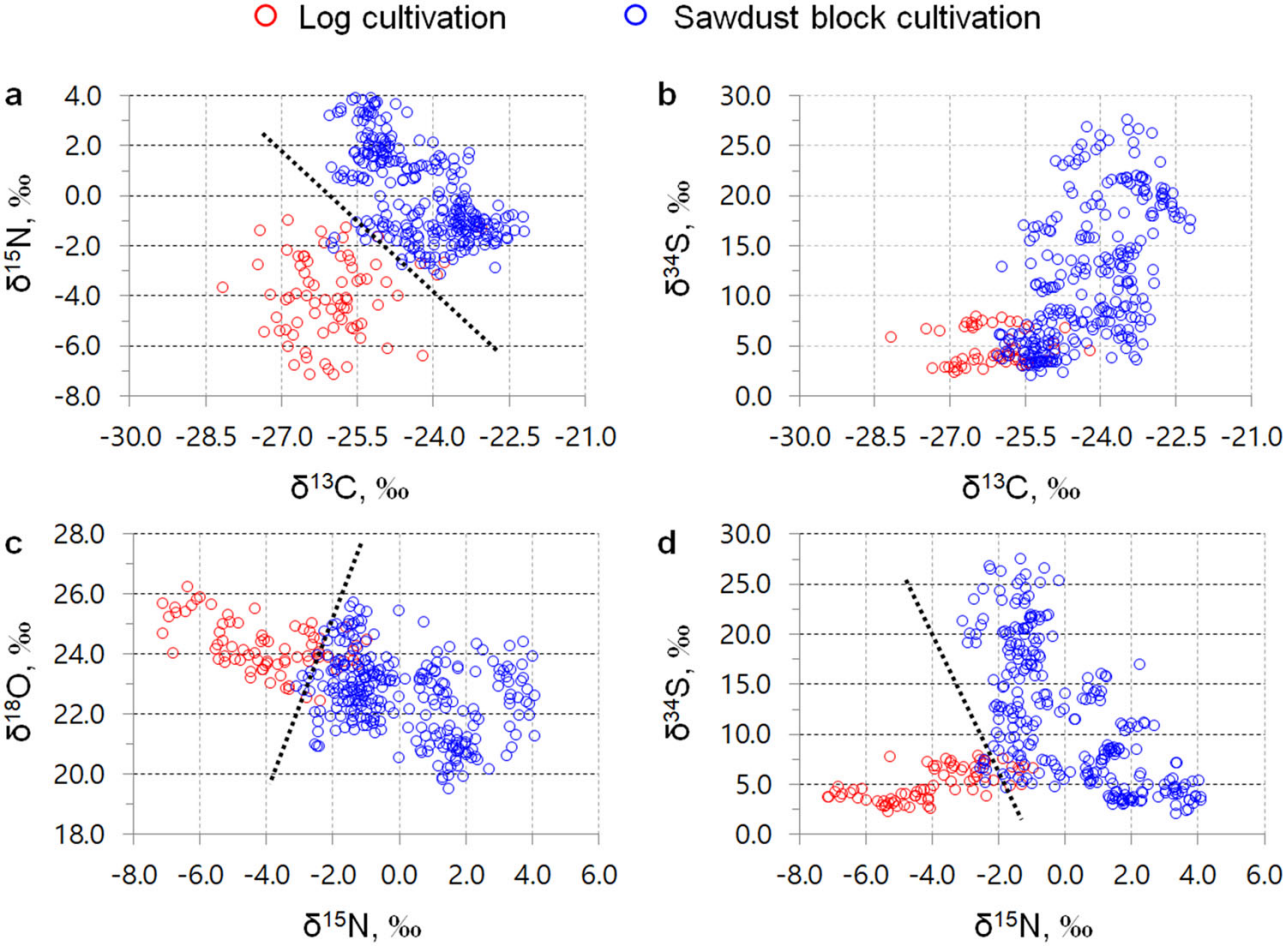
Two-dimensional (2D) plots of shiitake mushrooms produced using the log (red) and sawdust block (blue) methods. The dotted black lines are for illustrative purposes only and do not indicate the results of statistical analysis. The 2D plots by combinations of **a** δ^13^C and δ^15^N, **b** δ^13^C and δ^34^S, **c** δ^15^N and δ^18^O, and **d** δ^15^N and δ^34^S in shiitake.

**Fig. 2:**
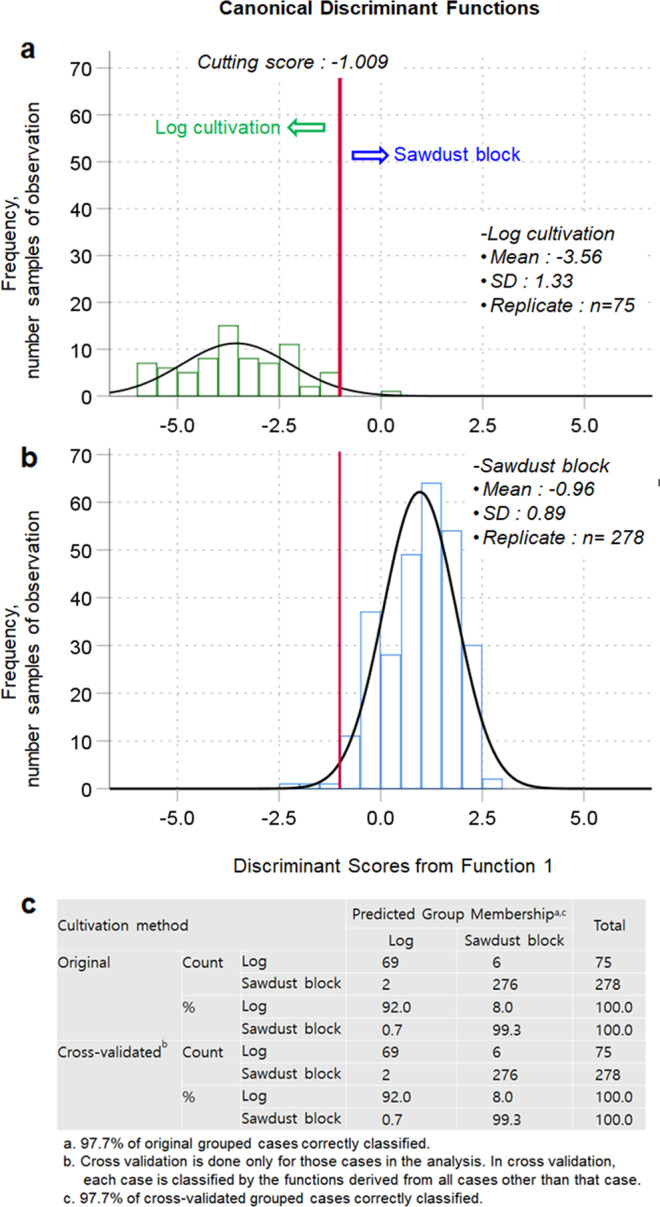
Discriminant analysis of shiitake mushroom depending on the cultivation method. **a** Discriminant scores of log cultivation, **b** Discriminant scores of sawdust block cultivation, and **c** Classification and cross-validated results of the shiitake cultivation method.

Table 1 shows the differences in δ^13^C, δ^15^N, δ^18^O, and δ^34^S in shiitake mushroom samples depending on the cultivation method (log versus sawdust blocks). Shiitake mushrooms produced using sawdust blocks included all three origins (Korea, Chinese inoculated sawdust block, and China). Owing to the availability of nutrients during the cultivation period, shiitake mushrooms prepared using sawdust blocks showed higher δ^13^C, δ^15^N, and δ^34^S, but lower δ^18^O, compared with shiitake mushrooms prepared using logs (*p* < 0.05). Next, two-dimensional (2D) plots of δ^13^C, δ^15^N, δ^18^O, and δ^34^S in shiitake mushrooms were used to visually assess discrimination of the shiitake mushroom cultivation method (Fig. 1). In particular, 2D plots related to δ^15^N showed a clear separation between log and sawdust block shiitake mushrooms (Fig. 1a–d). The discriminant function (*D* = [0.767 × δ^13^C] + [0.589 × δ^15^N] + [−0.130 × δ^18^O] + [0.079 × δ^34^S] + 21.532) demonstrated a clear separation between shiitake mushrooms prepared using the log and sawdust block cultivation methods based on a cutting score value of −1.009 calculated using the receiver operating characteristic (ROC) curve (Fig. 2a, b and Supplementary Tables 1 and 2). δ^15^N appeared to be the strongest predictor of the cultivation method according to the standardized canonical discriminant coefficient of 1.053. Additionally, the classification sensitivity and specificity based on ROC curves were 98.9% and 98.7%, respectively (Supplementary Tables 1 and 2). The overall classification accuracy by discriminant analysis (DA) was 97.7% for both the original set and the cross-validated group (Fig. 2c).

**Table 1:** Differences in stable isotope ratios (δ^13^C, δ^15^N, δ^18^O, δ^34^S) in shiitake mushrooms according to the geographical origin of the sawdust blocks and cultivation method (log *versus* sawdust block).

Cultivation method	δ^13^C	δ^15^N	δ^18^O	δ^34^S
Log (*n* = 75)	−26.01 ± 0.89^B^	−4.06 ± 1.60^B^	24.27 ± 0.83^A^	5.03 ±± 1.63^B^
Sawdust block (*n* = 279)	−24.28 ± 0.93^A^	0.08 ± 1.83^A^	22.66 ± 1.29^B^	11.94 ± 7.15^A^
LSD_0.05_	0.24	0.46	0.31	1.64
Sawdust block origin				
Korean origin (*n* = 125)	−24.95 ± 0.62^C^	1.70 ± 1.35^A^	22.15 ± 1.34^C^	7.85 ± 4.69^C^
Chinese inoculated (*n* = 94)^a^	−23.54 ± 0.68^A^	−1.20 ± 0.83^B^	23.34 ± 1.13^A^	16.77 ± 7.98^A^
Chinese origin (*n* = 60)	−24.02 ± 0.83^B^	−1.27 ± 0.96^B^	22.69 ± 0.86^B^	12.91 ± 4.55^B^
LSD *p* = _0.05_^b^	0.21	0.34	0.37	1.83

^a^The mean δ^18^O for shiitake mushrooms produced via the Chinese inoculated sawdust block method was based on the number of samples (*n* = 93).

^b^The value of LSD_*p* = 0.05_ is meant the results of the least significant difference test with the general linear model, which was performed at the 0.05 probability level.

^A–C^Data with different superscript capital letters are significantly different (*p* < 0.05) according to the LSD test.

### Discrimination of shiitake mushroom geographical origin

**Fig. 3:**
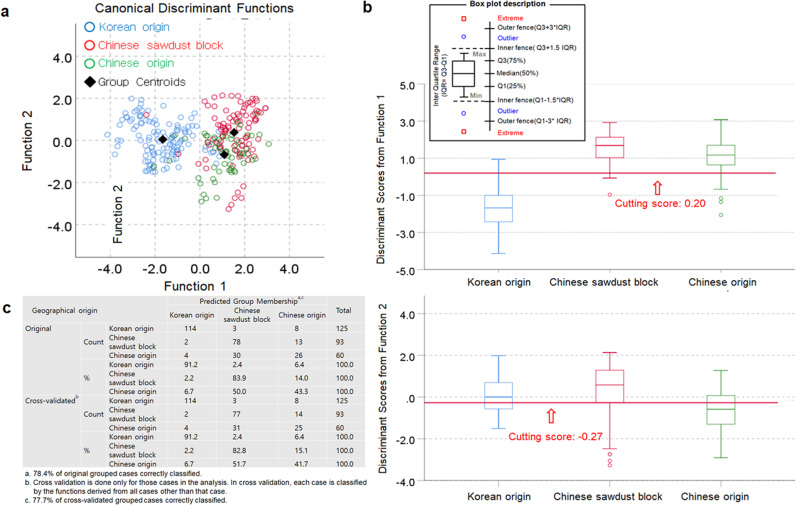
Discriminant analysis of the geographical origin of shiitake mushrooms according to the sawdust block source (Korean origin, Chinese inoculated sawdust block, Chinese origin). **a** Discriminant model developed by δ^13^C, δ^15^N, δ^18^O, and δ^34^S in shiitake mushrooms, **b** Box-and-whisker plots for discriminant scores derived from the first two canonical discriminant functions of the discriminant model developed for the geographical identification of shiitake mushrooms, and **c** Classification and cross-validated results of the origins of shiitake mushrooms produced by the different cultivation media.

Next, we examined the geographical origins of shiitake mushrooms obtained from Korea according to the sawdust block origin. The geographical discrimination model showed a clear separation between Korean origin and Chinese inoculated sawdust block/Chinese origin with a cutting score of 0.20 by the first canonical function, explaining 69.72% of the variation in the grouping variable, i.e., whether the mushrooms were of Korean origin. The second canonical function, with a cutting score of −0.27, could not effectively discriminate the origin of the shiitake mushrooms (Fig. 3a, b and Supplementary Tables 3 and 4). Consequently, this model showed insufficient classification accuracies of 78.4% for the original set and 77.7% for the cross-validated set. In particular, shiitake mushrooms of Chinese origin were identified as Chinese inoculated sawdust block shiitake mushrooms for both the original and cross-validated sets (Fig. 3c).

### Strategy for the identification of shiitake mushroom origin

**Fig. 4:**
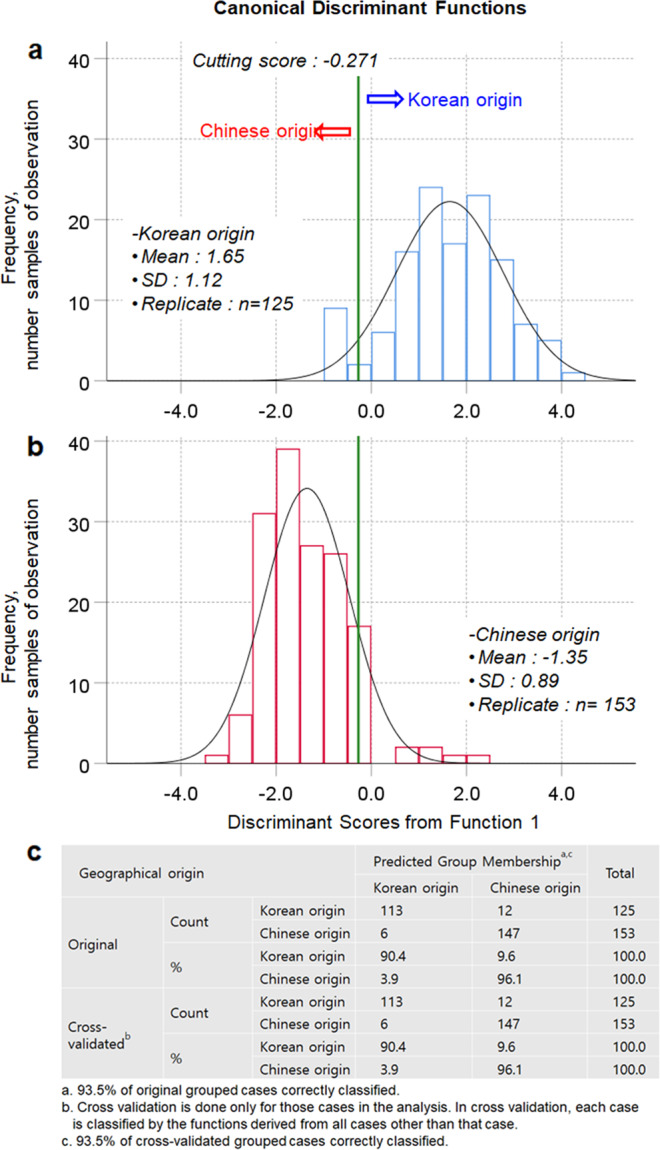
Discriminant analysis for the geographical identification of shiitake mushrooms produced using Chinese inoculated sawdust blocks, when considered as having a Chinese origin. **a** Discriminant scores for shiitake mushrooms with a Korean origin, **b** Discriminant scores for shiitake mushrooms with a Chinese origin, and **c** Classification and cross-validated results of the geographical origin of shiitake mushrooms.

**Fig. 5:**
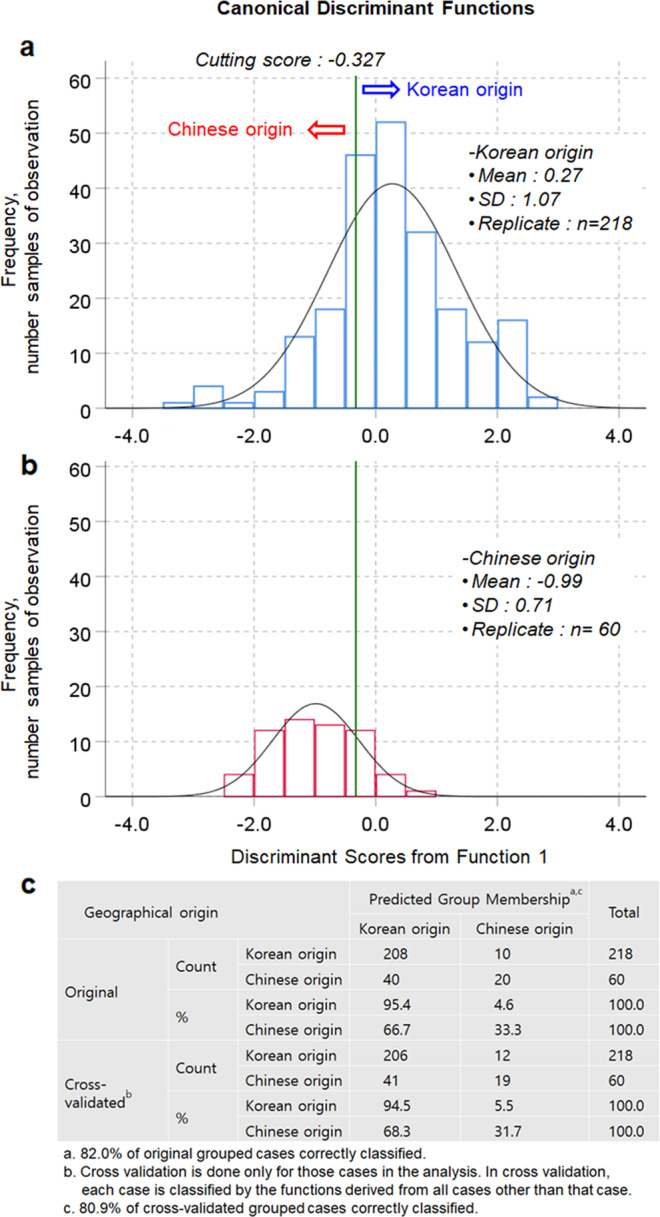
Discriminant analysis for the geographical identification of shiitake mushrooms produced using Chinese inoculated sawdust blocks, when considered as having a Korean origin. **a** Discriminant scores for shiitake mushrooms with a Korean origin, **b** Discriminant scores for shiitake mushrooms with a Chinese origin, and **c** Classification and cross-validated results of the geographical origin of shiitake mushrooms.

A new origin discrimination model was developed and evaluated for the identification of the geographical origin of shiitake mushrooms using the same dataset of stable isotope ratios considering two cases in which shiitake mushrooms generated using the Chinese inoculated sawdust block method were assumed as having Chinese origin (Fig. 4) or Korean origin (Fig. 5). In the first case, the geographical identification model showed a clear grouping between Korean and Chinese origin with a cutting score value of −0.271 by the first canonical function (*D* = [0.210 × δ^13^C] + [0.832 × δ^15^N] + [0.240 × δ^18^O] + [0.082 × δ^34^S] − 21.401). The canonical correlation of 0.831 showed that this model explained 69.06% of the variation in the grouping variable. The classification sensitivity and specificity based on the ROC curve were 92.0% and 91.5%, respectively (Supplementary Tables 5 and 6). The overall classification accuracy was 93.5% for both the original and cross-validated sample sets (Fig. 4a–c). Moreover, based on a standardized canonical discriminant coefficient of 0.793, δ^15^N appeared to be the most important predictor to discriminate whether the mushrooms were of Korean origin (Supplementary Tables 5 and 6).

In the second case, which considered shiitake mushrooms of Korean origin, the geographical identification model showed clear overlap between Korean and Chinese origin based on a cutting score value of −0.327 in the first canonical function, only explaining 21.25% of the variation in the grouping variable (Fig. 5a, b and Supplementary Tables 7 and 8). Consequently, this geographical discrimination model exhibited classification accuracies of 82.0% for the original sample set and 80.9% for the cross-validated sample set (Fig. 5c). Therefore, unlike the geographical identification of Korean origin according to the current labeling system, this approach was more accurate and suggested that the shiitake mushrooms produced using the Chinese inoculated sawdust blocks in Korea should be considered and labeled as having Chinese origin, despite the fact that the cultivation was carried out at many farms in Korea. Alternatively, it may be more suitable to provide labeling information establishing that the mushrooms were produced in Korea using sawdust blocks from China. Our results may also be applicable to other countries in which compost medium blocks are imported for other mushroom species such as *Agaricus bisporus*.

## Discussion

In this study, we evaluated the geographical origin identification of agricultural products and discussed the limitations of the current origin labeling system, particularly for cultivated mushroom species. In general, the country of origin is the country or region in which the agricultural products are produced or harvested. Internationally, the country of origin indicates the country in possession of the political entity in which the agricultural product was produced or harvested. Domestically, the country of origin also refers to a specific area or region within a country. The COOL system is permitted by international law and has been adopted by most countries (i.e., the EU, the USA, and Japan) to protect agricultural producers and consumers and to facilitate fair trade. Cases of illegal distribution of cheap, low-quality, foreign agricultural products disguised as domestic products have been reported in Korea, particularly during the initial period of trade liberalization by the World Trade Organization. Thus, in Korea, the COOL system was implemented in July 1991, and the enactment of the Act on the Origin of Agricultural and Marine Products for the unification of the origin labeling system occurred in 2010^22^.

To the best of our knowledge, compared with other foodstuffs, very few studies have described the geographical identification of shiitake mushrooms using SIRA. In a prior report based on δ^13^C and δ^15^N analyses^38^, 87.4% and 90.0% of Japanese dried shiitake mushrooms grown via log and mycelial cultivation (similar to sawdust blocks) could be discriminated from Chinese dried shiitake mushrooms grown via log and mycelial cultivation, respectively. However, the dried shiitake samples produced in Korea or Brazil via log or mycelial cultivation did not show suitable discriminative power against Japanese and Chinese dried shiitake mushrooms.

In addition, a recent model^39^ developed using SIRA (δ^13^C, δ^15^N, δ^18^O, δ^34^S) combined with orthogonal projection to latent structure-DA also showed good predictability (Q^2^Y = 0.892) for the geographical identification of dried shiitake slices between Korea and China; in particular, δ^15^N and δ^18^O were critical isotope indicators. In this prior report, the discriminative power for dried shiitake slices mainly resulted from the different cultivation methods between Korea (log cultivation) and China (sawdust block cultivation).

However, the identification of shiitake mushrooms originating from sawdust blocks, such as those of Korean origin, imported Chinese inoculated sawdust blocks (cultivated in Korea, the USA, or other countries), and of Chinese origin, has not been reported previously. Accordingly, origin labeling issues have recently emerged in various countries regarding shiitake mushrooms produced in Korea^16,39^ and the USA^17^ using Chinese inoculated sawdust blocks. Notably, for shiitake mushrooms produced via sawdust block cultivation, the origin labeling of shiitake mushrooms sold in Korea included information regarding the sawdust block source and cultivation region/country. However, because of some conflicts of interest between shiitake farms using domestic sawdust blocks and those using Chinese inoculated sawdust blocks, a revised origin labeling system that depends on the period of sawdust block preparation, inoculation, and cultivation in a specific country will be implemented in December 2020 in Korea^40^. Consequently, shiitake mushrooms prepared using Chinese inoculated sawdust blocks can be labeled as having a geographical origin of Korea or China depending on the period associated with shiitake mushroom production. This revised shiitake labeling system is still controversial among shiitake mushroom farmers using domestic or Chinese inoculated sawdust blocks. Similarly, shiitake mushrooms inoculated in China but produced in the USA are allowed to be labeled as “Product of USA.” Accordingly, the origin labeling of shiitake mushrooms produced using Chinese inoculated sawdust blocks is becoming a major issue worldwide^17^.

Our findings in this study clearly described the similarities and dissimilarities between shiitake mushrooms produced using Chinese inoculated sawdust blocks and those of Korean or Chinese origin. When mushrooms produced using Chinese inoculated sawdust blocks were assumed to have Chinese origin, the classification accuracy was 93.5%; this value dropped to 82% if the mushrooms were considered to have a Korean origin. Consequently, owing to similar SIR features, the geographical identification of shiitake mushrooms produced using Chinese inoculated sawdust blocks should first consider the country or region in which the sawdust blocks were produced. That is, the origin should be considered as China when Chinese inoculated sawdust blocks are used, regardless of the period of shiitake production in another country. Alternatively, a labeling system that includes both the origin of the sawdust blocks and the cultivation country/region may also be suitable to establish fair trade based on reliable origin labeling and to avoid conflicts with the rules of origin established by the World Trade Organization.

In conclusion, the origin labeling system for mushroom species, such as shiitake mushrooms, not soil-based products, must be discussed and appropriately revised to protect consumers and producers from fraud or mislabeling in the global market. At the same time, factors related to the COOL system must also be considered, as should methods to discriminate or label shiitake mushrooms originating from China or produced using Chinese inoculated sawdust blocks. This is the study reporting the limitations of the current COOL system for shiitake mushrooms in the global market. Additionally, our origin discrimination model based on stable isotope ratios may further improve the origin labeling of shiitake mushrooms to prevent fraud and contribute to fair international trade.

## Methods

The methods were performed in compliance with relevant guidelines and regulations and approved by the Faculty of Crop Science, Konkuk University.

### Shiitake mushroom collection

Fresh shiitake fruiting bodies (1 kg) were obtained from mushroom farms or retail markets in Korea from 2017 to 2019. Shiitake mushrooms produced via the sawdust block method were classified as Korean origin, Chinese inoculated sawdust blocks, and Chinese origin. Korean origin indicated that the mushrooms were produced using certain domestic sawdust blocks made by farms in Korea, and Chinese origin indicated that the mushrooms were fully produced in China. Mushrooms produced via the Chinese sawdust block method were defined as shiitake mushrooms produced in Korea using inoculated sawdust blocks imported from China. In addition, shiitake mushrooms produced via log cultivation were also obtained from some farms in Korea.

### Sample preparation for SIRA

The obtained mushrooms were lyophilized at −40 °C for 3 days and pulverized using a grinder to obtain a powder with particles smaller than 400 µm in size. For reliable δ^13^C, δ^15^N, δ^18^O, and δ^34^S measurements, ~2.5 mg was enclosed in a tin capsule (3.5 mm × 17 mm; IVA Analysentechinik e. K., Dusseldorf, Germany) for δ^13^C and δ^15^N, ~20 mg was added to a larger tin capsule (9 mm × 10 mm; Costech Analytical Technologies Inc., Valencia, CA, USA) for δ^34^S, and ~0.2 mg was added to a silver capsule (3.5 mm × 5.0 mm; Elemental Microanalysis, Okehampton, UK) for δ^18^O measurements. The encapsulated samples were placed in a desiccator until SIRA^41^.

### SIRA

δ^13^C and δ^15^N in shiitake mushrooms were simultaneously measured using a PDZ Europa ANCA-GSL elemental analyzer interfaced with a PDZ Europa 20-20 isotope ratio mass spectrometer (Sercon Ltd., Cheshire, UK)^29,41,42^. The encapsulated samples were first combusted in a reactor packed with tungsten (VI) oxide at 1000 °C. Subsequently, oxide products were removed in a reduction reactor using reduced copper at 650 °C. Next, the remaining sample gas flowed through a water trap composed of magnesium perchlorate and phosphorous pentoxide along with a helium carrier gas. Finally, CO_2_ was retained on an adsorption trap until the N_2_ peak was analyzed; subsequently the adsorption trap was heated at 165 °C to release CO_2_ and then analyzed via isotope ratio mass spectrometry (IRMS).

δ^18^O in shiitake mushrooms was analyzed using an Elementar PyroCube (Elementar Analysensysteme GmbH, Hanau, Germany) interfaced to an Isoprime VisION (Isoprime Ltd., Stockport, UK, a subsidiary of Elementar Analysensysteme GmbH, Hanau, Germany). The enclosed samples were thermally decomposed to CO in a glassy carbon reactor at 1400 °C. Next, the CO was isolated using an adsorption trap from any interfering gas, such as N_2_, and subsequently subjected to the IRMS system.

δ^34^S in shiitake mushrooms was measured using an Elementar Vario ISOTOPE cube interfaced to a SerCon 20-22 IRMS. The encapsulated samples were first combusted in a reactor with tungsten oxide at 1150 °C. Thereafter, sample gases were reduced using elemental copper at 880 °C and then passed through a buffering reactor at 900 °C. Next, SO_2_ and CO_2_ were separated by purge and trap, which enabled full separation and peak focusing. Finally, the adsorption trap was heated, and the sample SO_2_ was released into the IRMS for δ^34^S measurement.

Provisional isotope ratio values were adjusted and corrected against the laboratory reference materials (RMs), and the δ^13^C, δ^15^N, δ^18^O, and δ^34^S values in the shiitake mushroom samples were finally calculated in part per thousands (‰) with δ value notation: δ, ‰ = ([*R*_sample_ – *R*_standard_]/*R*_standard_) × 1000, where *R* is each stable isotope ratio of the sample of interest and the international reference standards. Vienna PeeDee Belemnite for carbon (^13^C/^12^C), atmospheric N_2_ for nitrogen (^15^N/^14^N), Vienna Standard Mean Ocean Water for oxygen (^18^O/^16^O), and Vienna Canyon Diablo Troilite for sulfur (^34^S/^32^S) were used as the international reference standards^43^.

Several laboratory RM replicates, which showed an isotopic composition similar to that of the shiitake mushroom samples, were also analyzed for reliable δ^13^C, δ^15^N, δ^18^O, and δ^34^S measurements along with the samples. The laboratory RMs had been calibrated against international RMs and were used for the evaluation of long-term analytical precision described as standard deviations as follows: ≤±0.11‰ for δ^13^C, ≤±0.12‰ for δ^15^N, ≤±0.24‰ for δ^18^O, and ≤±0.37‰ for δ^34^S during this study period.

### Statistical analysis

In this study, more than five analytical replicates (*n* ≥ 5) of shiitake mushrooms obtained from each farm or retail market were used for the δ^13^C, δ^15^N, δ^18^O, and δ^34^S analyses. Each replicate was prepared by pulverization and pooling of at least five shiitake fruiting bodies from all entire samples (1 kg, farm or market). Statistical analysis was first conducted using the least significant difference test with the general linear model, which was performed at the 0.05 probability level using the statistical analysis program SAS (version 9.2; SAS Institute Inc., Cary, NC, USA). The results were reported as the mean ± standard deviation of each measurement. Next, for unequally sized grouping variables (shiitake mushroom origin or cultivation method), all independents (i.e., δ^13^C, δ^15^N, δ^18^O, and δ^34^S) were applied to DA with Wilks’s Lamda method. A cutting score for the binary classifier system was calculated using ROC curves (Supplementary Tables 2, 6, and 8) or was calculated considering the group size and centroid values as follows: *Z*_cutting_ = (*N*_a_ × *Z*_b_ + *N*_b_ × *Z*_a_)/(*N*_a_ + *N*_b_), where *N*_a_ and *N*_b_ are the sizes of groups a and b, and *Z*_a_ and *Z*_b_ are the centroids of groups a and b (Supplementary Table 4)^44^. ROC curves were also used for a more complete evaluation of the classification sensitivity and specificity. All discrimination models (i.e., origin and cultivation method) developed in this study were evaluated for the classification accuracy of the cross-validated set for unknown samples using the leave-one-out method (IBM SPSS statistics version 24, Armonk, NY, USA).

## Supplementary information

Supplementary Tables 1–8

Dataset

reporting-summary

## Data Availability

The datasets generated during and/or analyzed during the current study are available in the [figshare] repository, [10.6084/m9.figshare.13371827].
