# Supplementary material for: An origin identification model for labeling of shiitake (Lentinula edodes)
Source: NPJ Sci Food. 2021 Feb 2;5:2. doi: 10.1038/s41538-021-00085-4 (PMC7854607; doi:10.1038/s41538-021-00085-4)
Supplement: Supplementary file 3 — reporting-summary [file 41538_2021_85_MOESM3_ESM.pdf]

nature research

Corresponding author(s): Seung-Hyun Kim

Last updated by author(s): Dec 14, 2020

Reporting Summary

Nature Research wishes to improve the reproducibility of the work that we publish. This form provides structure for consistency and transparency in reporting. For further information on Nature Research policies, see our Editorial Policies and the Editorial Policy Checklist.

Statistics

For all statistical analyses, confirm that the following items are present in the figure legend, table legend, main text, or Methods section.

n/a	Confirmed	
☐	☒	The exact sample size (*n*) for each experimental group/condition, given as a discrete number and unit of measurement
☐	☒	A statement on whether measurements were taken from distinct samples or whether the same sample was measured repeatedly
☐	☒	The statistical test(s) used AND whether they are one- or two-sided<br>*Only common tests should be described solely by name; describe more complex techniques in the Methods section.*
☐	☒	A description of all covariates tested
☐	☒	A description of any assumptions or corrections, such as tests of normality and adjustment for multiple comparisons
☐	☒	A full description of the statistical parameters including central tendency (e.g. means) or other basic estimates (e.g. regression coefficient) AND variation (e.g. standard deviation) or associated estimates of uncertainty (e.g. confidence intervals)
☐	☒	For null hypothesis testing, the test statistic (e.g. F, t, r) with confidence intervals, effect sizes, degrees of freedom and P value noted<br>*Give P values as exact values whenever suitable.*
☒	☐	For Bayesian analysis, information on the choice of priors and Markov chain Monte Carlo settings
☒	☐	For hierarchical and complex designs, identification of the appropriate level for tests and full reporting of outcomes
☒	☐	Estimates of effect sizes (e.g. Cohen's d, Pearson's r), indicating how they were calculated

Our web collection on statistics for biologists contains articles on many of the points above.

Software and code

Policy information about availability of computer code

Data collection	Data were collected by the Callisto (version 8.40) and IonOS 4.3 software.
Data analysis	All data were analyzed by SAS (version 9.2) and IBM SPSS statistics (version 24).

For manuscripts utilizing custom algorithms or software that are central to the research but not yet described in published literature, software must be made available to editors and reviewers. We strongly encourage code deposition in a community repository (e.g. GitHub). See the Nature Research guidelines for submitting code & software for further information.

Data

Policy information about availability of data

All manuscripts must include a data availability statement. This statement should provide the following information, where applicable:

- Accession codes, unique identifiers, or web links for publicly available datasets
- A list of figures that have associated raw data
- A description of any restrictions on data availability

The datasets generated during and/or analyzed during the current study are available in the [figshare] repository, [DOI: 10.6084/m9.figshare.13371827].

Field-specific reporting

Life sciences study design

All studies must disclose on these points even when the disclosure is negative.

Sample size	Fresh shiitake fruiting bodies (1 kg) were obtained from mushroom farms (n=27) or retail markets (n=7) in Korea from 2017 to 2019.
Data exclusions	No data were excluded from the study.
Replication	More than five replicates ($n \geq 5$) of shiitake mushrooms obtained from each farm or retail market were used for the δ13C, δ15N, δ18O, and δ34S analyses.
Randomization	Each replicate was prepared by pulverization and pooling of at least five shiitake fruiting bodies from all entire samples (1 kg, farm or market).
Blinding	This study is non-clinical study, so blinding was not applicable.

Reporting for specific materials, systems and methods

We require information from authors about some types of materials, experimental systems and methods used in many studies. Here, indicate whether each material, system or method listed is relevant to your study. If you are not sure if a list item applies to your research, read the appropriate section before selecting a response.

Materials & experimental systems

n/a	Involved in the study	
☒	☐	Antibodies
☒	☐	Eukaryotic cell lines
☒	☐	Palaeontology and archaeology
☐	☒	Animals and other organisms
☒	☐	Human research participants
☒	☐	Clinical data
☒	☐	Dual use research of concern

Methods

n/a	Involved in the study	
☒	☐	ChIP-seq
☒	☐	Flow cytometry
☒	☐	MRI-based neuroimaging

Animals and other organisms

Policy information about studies involving animals; ARRIVE guidelines recommended for reporting animal research

Laboratory animals	This study did not include laboratory animals.
Wild animals	This study did not include wild animals.
Field-collected samples	This study did not include the field-collected samples.
Ethics oversight	Ethical approval was not necessary for this study because this study included commercially produced shiitake mushroom samples.

Note that full information on the approval of the study protocol must also be provided in the manuscript.
